# Impact of disturbed areas on Theraphosidae spiders diversity (Araneae) and first population data of *Grammostola rosea* (Walckenaer) in Panul Park

**DOI:** 10.1002/ece3.5163

**Published:** 2019-05-08

**Authors:** Milenko A. Aguilera, Rubén Montenegro V., María Eugenia Casanueva

**Affiliations:** ^1^ Facultad de Ciencias Naturales y Oceanográficas, Departamento de Zoología Universidad de Concepción Concepción Chile; ^2^ Aracno Inc. S.p.A. Investigaciones Científicas y Ambientales Concepción Chile; ^3^ National Museum of Natural History Santiago Metropolitan Region Chile

**Keywords:** anthropic disturbance, anthropogenic impact, chilean tarantula, methodology environmental baseline

## Abstract

Soil fauna constitutes one of the most abundant and richest environments on earth (Coleman et al. 2004, Fundamentals of solil ecology, 2nd ed. Elsevier Academic Press, London, UK). Different degrees of soil disturbance can affect arthropod diversity, which allows a correlation of biodiversity to quality of habitat. The present study aimed to evaluate the impact of habitat on Theraphosidae spiders, with special focus on *Grammostola rosea*. Slight differences in the diversity of Theraphosidae between the disturbed area of Cerro Huechuraba and the undisturbed Panul Park were found. However, a high dominance of *G. rosea* was observed in both study areas. *G. rosea* density 1,350 ind/ha in Panul Park, and 750 ind/ha in Cerro Huechuraba. UPGMA cluster analysis did not show significant differences between established environments. A standard methodology to develop inventories of Theraphosidae was proposed. The distribution of *G. rosea* and its natural history were reported.

## 1 INTRODUCTION

Soil is one of the habitats with the greatest species richness on earth, but also one of the least studied (Decaëns, Jiménez, Gioia, Measey, & Lavelle, 2006; Giller, Beare, Lavelle, Izac, & Swift, 1997; Wolters, 2001). Arthropods represent 97% of soil fauna, and 12% of this corresponds to arachnids (Decaëns et al., 2006). Arthropod diversity can vary depending on degree of environmental disturbance, thereby indicating a correlation of biodiversity to quality of habitat (Duelli & Obrist, 1998). Spiders, considered as hyperdiverse groups, are very common in terrestrial ecosystems and have great ecological diversity (Jimenez‐Valverde & Hortal, 2003). Among the studies of spiders are those dealing with their potential to control pests by depredation (Greenstone & Sunderland, 1999; Harwood, Sunderland, & Symondson, 2004; Maloney, Drummond, & Alford, 2003; Nyffeler & Sunderland, 2003), with guild structure (Cardoso, Pekár, Jocqué, & Coddington, 2011; Uetz, Halaj, & Cady, 1999), with diversity, distribution, fragmentation, and competition effects (Torma, Gallé, & Bozsó, 2014), and with bioindicators of soil quality (Marc, Canard, & Ysnel, 1999; Willett, 2001). Given their prominence, several authors have studied the effect and relative importance of local environmental factors on spiders and other arachnids (Lo‐Man‐Hung et al., 2011).

However, studies of the biology, ecology, and taxonomy of spiders have been delayed compared to other groups (Bertani, 2001; Jimenez‐Valverde & Hortal, 2003; Marc et al., 1999). Even more scarce are those studies in tarantulas, mainly due to their way of life and the obtaining specimens (Ferretti, Schwerdt, Peralta, Farina, & Pompozzi, 2016).

Among the Theraphosidae present in Chile, *Grammostola rosea* (Walckenaer, 1837) is the most frequently mentioned in informal reports, but no data about its population distribution are given. Moreover, historical distributions may no longer be useful because humans in their imperative need for expansion have notoriously modified the environment. Consequently, land use has shifted to urban use along with alterations of vegetation and geography (Argañaraz & Gleiser, 2017), thereby producing local changes in arachnid diversity. In addition, some historical distributional data may be wrong, as is the case with *G. rosea*, which was cited for Mexico, a labeling error by Pickard‐Cambridge (1897) detected by Pocock (1903).

Another important factor is that wild specimens of this species are under great capture pressure to be sold abroad as pets, which could eventually cause a drastic decline in populations and possibly local extinctions (Aguilera, 2015; ODEPA, 2017).

The present study aim was to evaluate habitat impacts on communal and distributional parameters of *Grammostola rosea*, in two areas with different levels of anthropic disturbance and land use, and especially in an ecological preservation area. Furthermore, data related to habitat and natural history of the species, proposed methodologies to correctly evaluate *Grammostola* populations in environmental baseline are given.

## 2 MATERIALS AND METHODS

### 2.1 Study area

**Figure 1:**
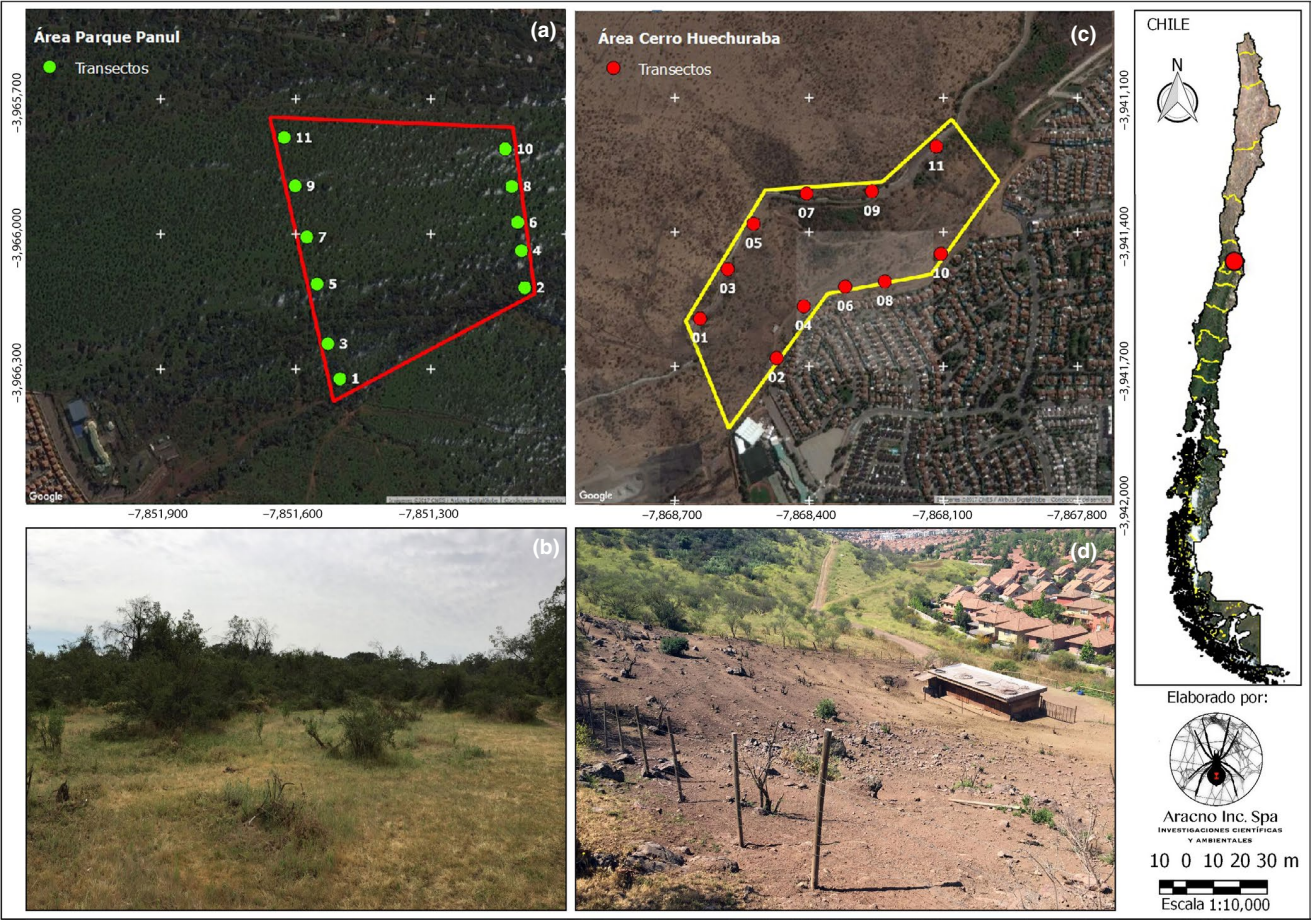
(a) Cartography of Panul Park study area. (b) Panul Park study area, annual Forest and Poaceae formation. (c) Cartography of Cerro Huechuraba study area. (d) Cerro Huechuraba study area, from top to bottom formation of wild allochthonous, Forest and devoid of vegetation with poaceas

Forest of Panul Park, with an approximate sampling area of 13.5 ha (Figure 1a) in the eastern sector of La Florida (357,928 E, 6,288,800 S). Located in the foothills of one of the closest mountain ranges of Santiago, known as the Sierra de Ramón, where the highest elevations exceed 3,000 m above sea level. Recently, this sector has had an economic interest for real estate development, and therefore, some environmental studies have been performed in the area (Gesterra, 2011), but none about arachnids. Due to the importance of the area and the imminent urbanization, the Corporación Municipal de Fomento al Desarrollo Comunal y Productivo of La Florida (COFODEP, 2015) suggests Panul Park as an “environmental ecological preservation area of the foothills, municipality of La Florida.”

Panul Park is known as the last native sclerophyllous forest in Santiago, alternating secondary sclerophyllous scrubs (Mann, 1964; Figure 1b). In this study area, two environments were characterized: a Forest composed of sclerophyllous forest patches and scrubs of *Acacia caven* (Molina) Molina and a second environment devoid of arboreal vegetation and covered mainly by annual poaceae and isolated specimens of *A. caven*.

The second study area was located in Cerro Huechuraba (343,367 E, 6,308,994 S) with an approximate inspection area of 13 ha (Figure 1c); at 3 km north of Route 70 (in the 1,700 block of Américo Vespucio Ave.) and 1.5 km east of Route 52 (in the 7,500 block of San Martin Ave.) toward the north of the Huechuraba urban zone, defined as an urban housing area according to the Communal Regulatory Plan. This last area has an important anthropic intervention, due to the presence of houses in the periphery and stables, grazing areas in the study area. Only this last one has flora and wild fauna. In this area, three environments were observed: Forest formed by small vegetational patches of *A. caven*, a second environment of wild allochthonous of dense patches of *Rubus ulmifolius* Schott 1818, *Loasa tricolor* Ker Gawl. 1822 and *A. caven,* and a third environment laking arboreal vegetation and covered mainly by annual poaceae (Figure 1d).

Geographical coordinates are expressed in UTM WGS84 19H.

### 2.2 Sampling

Collection was performed during the spring of November 2016, using stratified random sampling with eleven transects of 200 m length by 2 m width, with a pedestrian path at low speed for two specialists in each area for a minimum of 30 min by transect. To confirm inhabitation of burrows, a flexible endoscope with a video microcamera with a lighting source connected by fiber optic to a screen, which allowed insertion of the fiber optic cable inside the burrow for inspection, was used. A manual collection was performed (Sorensen, Coddington, & Schaft, 2002; Upton, 1991), and the individuals were stored in 95% ethanol prior to labeling, and later deposited in the arachnological collection of the Museum of Zoology of the University of Concepción (MZUC‐UCCC), Concepción, Chile and National Museum of Natural History (MNHN), Santiago, Chile. Collects were authorized by the Servicio Agrícola y Ganadero, Exempt Resolution No. 6382/2016 Metropolitan SAG.

In order to obtain data of the biology and current distribution of *G. rosea*, additional collects and observations were made throughout the potential distribution of the species. (29°S and 40°S) in Chile, between 2016 and 2018, and the collections of MNHN and UCCC‐UDEC were revised (see Data accessibility).

### 2.3 Breeding

30 specimens were raised in glass boxes of 30 × 30 × 30 cm. Artificial burrows attached to glass were built for observation. All containers had a substrate of soil, *Sphagnum* sp. and water provision. They were fed once every 2 weeks mainly with cockroaches (*Blatta* sp.), crickets (Gryllidae), *Tenebrio* sp., and *Zophobas* sp. Temperature in laboratory varied between 16°C and 34°C. Behaviors were studied by direct observations and registered by notes and photographs.

### 2.4 Taxonomy

For identification of theraphosids, the morphology of the tibial processes by Montes de Oca, D'Elía, and Pérez‐Miles (2016) and Montenegro, Aguilera, and Casanueva (2018), the palpal bulb structure by Bertani (2000), the spermatheca morphology by Schiapelli and Gerschman (1961), and the urticating setae by Cooke, Roth, and Miller (1972) were used. Identifying images were obtained with a stereoscopic magnifier Motic SMZ‐140 and ZeissStemi SR with its supplement for a Nikon Coolpix P600 camera.

### 2.5 Statistics

In order to quantify *Grammostola rosea* and the other Theraphosidae in the study sites, a diversity analysis was performed using the computer program PAST 3.16 (Hammer, Harper, & Ryan, 2001) to determine S richness, *H*′ diversity according to Shannon–Weaver (Shannon & Weaver, 1949), and *J*′ uniformity according to Simpson (1949). The Shannon–Weaver and Simpson indices were used because they allow comparisons of Araneofauna in terms of richness proportional to abundance, and evenness of abundance among the species in the two study areas. Moreover, these indices are frequently used in the literature (Jaksic, 2001) and therefore facilitate comparisons with similar studies (Aguilera, Casanueva, & Hernández, 2006). In order to compare alpha biodiversity estimators between both study sites, a comparison test was performed with 1,000 iterations in the PAST program, based on the bootstrapping technique (Manly, 1997). To evaluate the degree of similarity of tarantulas between the two study areas, the Jaccard similarity coefficient was used based on presence/absence taxa records. In order to evaluate the existence of groupings according to localities and/or vegetation formation, a cluster analysis was performed using Jaccard similarity values. For this conglomerate, the abundance data of each transect were analyzed and the unweighted pair‐group method using arithmetic averages (UPGMA) was used (Sokal & Rohlf, 1995). Species accumulation curve was calculated (Collwel & Coddington, 1994; Jimenez‐Valverde & Hortal, 2003) to evaluate the information collected in the field and also to determine the efficiency of the characterization of the influence area. Additionally, density of species per m^2^ was also estimated for each study area.

## 3 RESULTS

A total of 86 individuals were recorded from the transect sampling, 30 in the Huechuraba area and 56 in Panul Park, belonging to *Grammostola rosea*, *Homoeomma chilensis* Montenegro & Aguilera, 2018 and one *Euathlus* sp. (Table 1). In the Huechuraba area, only *G. rosea* and *Euathlus* sp. were recorded with a relative abundance of 96.7% and 3.3%, respectively, while in Panul Park, 96.4% abundance of *G. rosea* was observed, and *Euathlus* sp. and *Homoeomma chilensis*. represented 1.8% each with an estimated *G. rosea* density of 1,350 ind/ha for Panul Park, and 750 ind/ha for Cerro Huechuraba.

**Table 1:** Number of individuals per study area and environments

Environment	Transect	*Grammostola rosea*	*Euathlus* sp.	*Homoeomma* sp.
Forest	P1	10	0	0
Forest	P2	0	0	0
Forest	P3	2	0	0
Forest	P4	18	0	0
Scrub/Poaceas	P5	4	0	0
Scrub/Poaceas	P6	0	0	0
Scrub/Poaceas	P7	0	0	0
Scrub/Poaceas	P8	9	0	1
Scrub/Poaceas	P9	4	1	0
Scrub/Poaceas	P10	0	0	0
Scrub/Poaceas	P11	7	0	0
Forest	H1	4	0	0
Forest	H2	4	0	0
Scrub/Poaceas	H3	3	0	0
Scrub/Poaceas	H4	3	0	0
Allochthonous	H5	0	0	0
Allochthonous	H6	1	0	0
Scrub/Poaceas	H7	3	0	0
Scrub/Poaceas	H8	6	0	0
Scrub/Poaceas	H9	2	0	0
Forest	H10	3	1	0
Allochthonous	H11	0	0	0

Note

H: Cerro Huechuraba; P: Panul Park.

**Figure 2:**
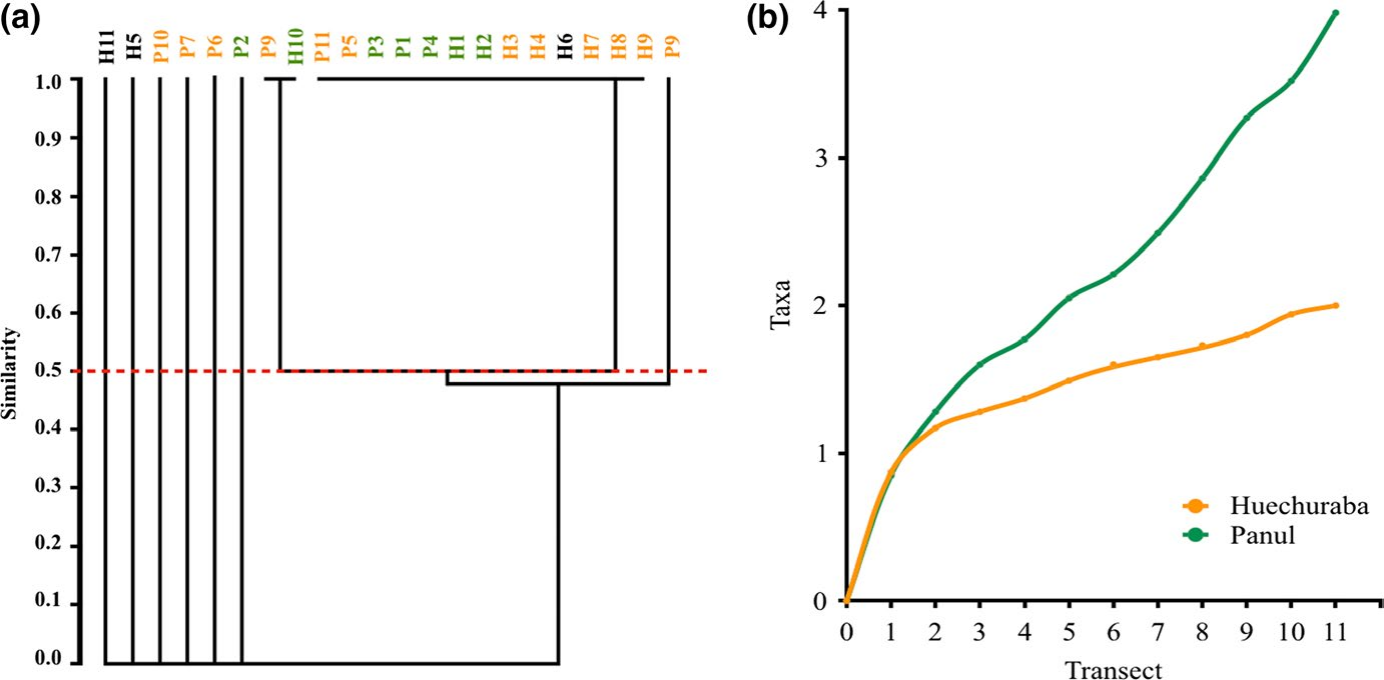
(a) Similarity phenogram obtained from UPGMA analysis based on presence or absence of theraphosid spider within the two study areas. H: Cerro Huechuraba; P: Panul Park; Green, Forest; Orange, Scrub and poaceas; Black, allochthonous (b) Species accumulation curve for both sampling areas

In relation to the communal parameters (Table 2), it can be observed that Panul Park had a greater *H*′ diversity (*H* = 0.178) compared to Huechuraba (*H* = 0.146), but without significant statistical differences, while the J’ index indicates that the abundances are similarly distributed in both study areas, but since the *J*′ values are considerably low indicate a dominance given by *G. rosea*. On the other hand, *G. rosea* density values are greater in Panul Park than in Huechuraba. The cluster analysis (UPGMA + Jaccard) evaluated Theraphosidae similarity between the two study sites and did not show significant groupings according to environments, or they would be expected to occur by chance (Figure 2a). In the species accumulation analysis, it was observed that the asymptotic number of species was not reached in any of the two study areas, so objective criteria cannot be applied to determine whether the species inventory was sufficiently complete (Figure 2b).

**Table 2:** Alpha biodiversity estimators in the two study areas

Alfa biodiversity	Panul Park	Cerro Huechuraba
Richness S	3 (2–3)	2 (2–2)
Shannon H	0.18 (0.08–0.36)	0.14 (0.14–0.39)
Simpson J	0.06 (0.03–0.16)	0.06 (0.06–0.23)
Density (m^2^) *G. rosea*	0.135	0.073
Density (m^2^) *Euathlus* sp.	0.003	0.003
Density (m^2^) *Homoeomma cf chilensis*	0.003	0

Note

Parentheses indicate the lower and upper limits of confidence intervals calculated from a bootstrap process with 1,000 replicas

**Figure 3:**
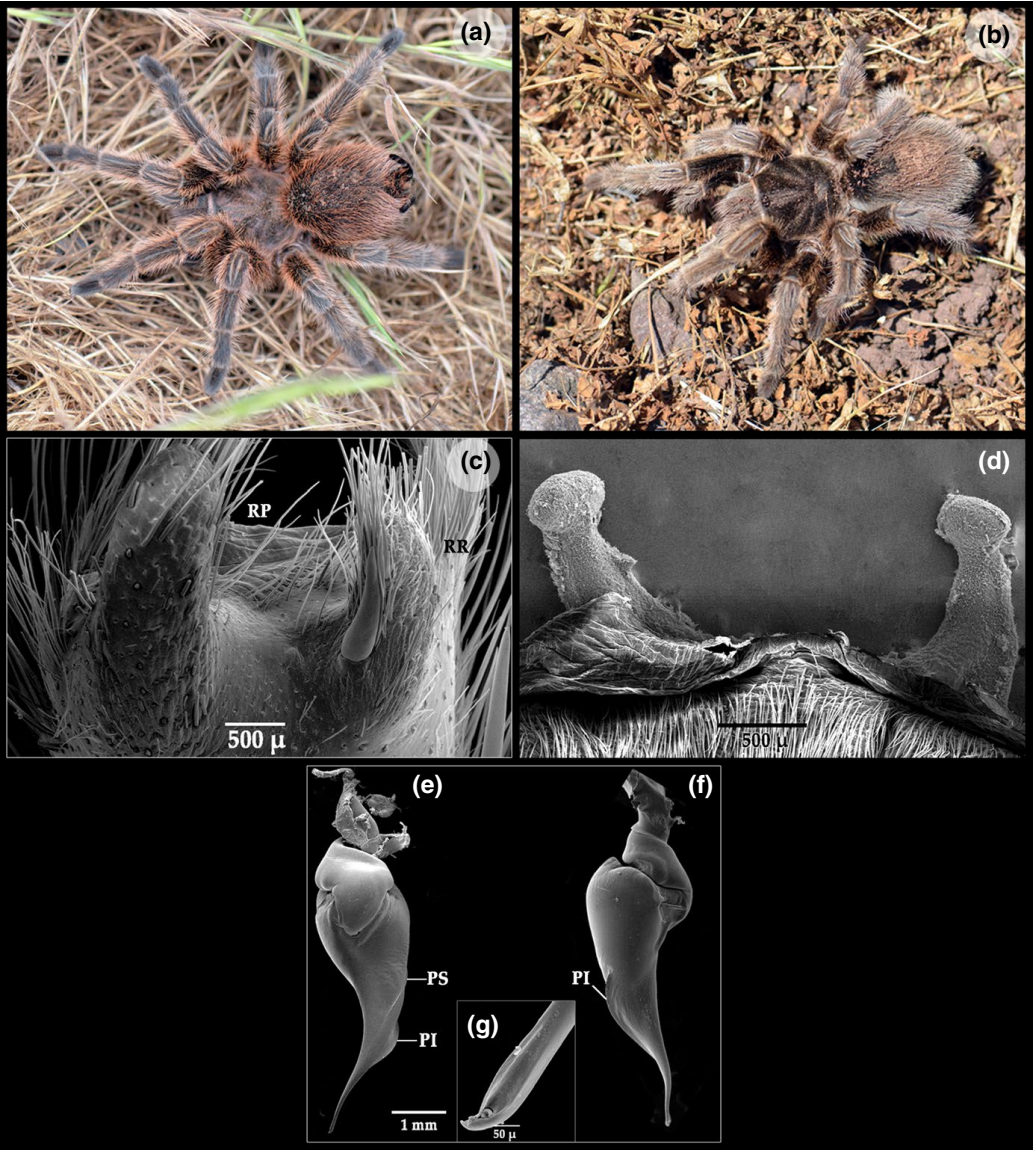
*Grammostola rosea*. (a) rose variety. (b) brown variety. (c) Tibiae I apophysis, ventral view. (d) spermathecae. (e) right palp, retrolateral view. (f) right palp, prolateral view. (g) embolus apex, detail. LP: Lower prolateral keel; PB: prolateral branch; RB: retrolateral branch; UP: Upper prolateral keel

### 3.1 Natural history

*Grammostola rosea* (Figure 1a–g). This species lives at low altitudes, ranging between 0 and 1,500 m above sea level, with fragmented populations distributed between 28.5°S (Vallenar, Atacama Region) and 37.8°S (Angol, Biobio Region) (see data accessibility). In the field, it has been observed that males of *G. rosea* are living in burrows built directly into the ground with little stony substrates, between undergrowth and under large stones. Females build burrows of different depths, reaching a maximum of 40 cm. Generally, the burrows are a straight tunnel with one chamber at the bottom and sometimes a second chamber at two thirds of depth. The entrance frequently covered with silk. Sexual activity periods were estimated through the presence of living mature males in the field during the study period and occur mainly between September and March (spring and summer seasons in the Southern Hemisphere). A second activity period occurs between May and July. Egg‐sacs of *G. rosea* were observed throughout December to February.

In Laboratory, two females copulated in June. Six months after copulation (December), the females perform egg‐sac oviposition and spiderlings emerged 60 days after (February). As average, a total of 253 spiderlings were counted.

Regarding diet, this species consumes crickets of various species from the Gryllidae family as well as beetles, mainly Scarabidae. For predators, it was possible to observe wasps from the family Pompilidae (*Pepsis* sp. and *Pompilocalus* sp.), hunting spiders, and small lizards such as *Liolaemus lemiscatus* Gravenhorst (1838).

It has been observed that *G. rosea* has various color patterns, frequently with reddish to dark brown tones in the setae, cephalothorax, and abdomen (Figure 1a,b). Additionally, the characters of the spermatheca of the female, bulb, and tibial apophysis of the male for *G. rosea* are given in Figure 1c–g.

In particular in the areas of study, high burrow density was observed for some of the sectors sampled, with distances of no more than 1 m between burrows. In Panul Park, burrows were found among different vegetational formations, both in sclerophyllous forests and in environments devoid of vegetation or with annual isolated individual poaceae and *A. caven*, while in Cerro Huechuraba burrows were mainly found in vegetational formations of forest and scrub.

## 4 DISCUSSION

Theraphosidae spiders inhabiting areas with various degrees of anthropic disturbance respond in different ways to habitat changes, especially because they depend on the landscape for food and shelter (Goncalves‐Souza, Matallana, & Brescovit, 2008). This study showed theraphosid communities vary slightly according to conservation status of the area, which mainly affected richness and abundance of Theraphosidae, in alignment with Goncalves‐Souza et al. (2008). Comparison of diversity indices pointed to a slightly higher diversity in the Panul forest area, along with a marked dominance of *G. rosea*, accounting for 96% of the samples obtained. *G. rosea*, although sympatric with the found other two Theraphosidae species were dominant in the study areas, perhaps due to its way of life and the micro environmental requirements needed for its development. Notably, this species can build its burrow directly on the ground, unlike *Euathlus* and *Homoeomma chilensis*, which require a stony substrate for their burrows. It is that *G. rose*a has larger number of offspring than *Euathlus* and *H. chilensis* (Aguilera, 2015) and therefore explains the higher population density. Goncalves‐Souza et al. (2008) indicated that specific environmental factors such as microhabitats, closed vegetation as protection against predators, temperatures, humidity, and light intensity can explain variations of spider diversity among different habitats.

Diversity differed slightly between the two study areas (see *H*′ and *J*′ indices), with a notable abundance of *G. rosea*, although 46% lower in Cerro Huechuraba (anthropically intervened) than in Panul Forest. In both study areas, no significant cluster was observed when considering the present environments (see UPGMA analysis). This showed high similarity between the environments; therefore, these would behave as habitat units structurally homogeneous for the spiders, and *G. rosea* would not be restricted by the types of environments present (forest/scrub and cover of poaceas).

Despite this, in Cerro Huechuraba, the relative abundances were considerably lower in wild allochthonous environment than in the forest/scrub and cover of poaceas environments. Furthermore, density followed the same pattern indicated for relative abundance and was estimated at 1,350 ind/ha for Panul Park, and 750 ind/ha for Cerro Huechuraba.

As for the obtained species accumulation curve, it was not possible to reach asymptotic numbers of species. In this context, there are no objective criteria that determine when the Theraphosidae inventory in both study areas is sufficiently complete. As reported by Jimenez‐Valverde and Hortal (2003), the fact that it was not possible to reach asymptotic numbers could be due to temporal or spatial biases in the sampling effort distribution that could affect the curve shape. In the present study, collection was performed during spring; therefore, temporal bias was considered as spring and to a lesser extent summer is the ideal sampling times for Theraphosidae. This is on account of its seasonal life cycles, and these times correspond with a higher activity cycle, including reproductive and growing seasons (Aguilera, 2015). During these seasons, adult males and females are more easily found, along with younger populations (personal observations), therefore facilitating proper estimation of Theraphosidae population densities. Additionally, in order to avoid generating spatial bias of the sampling effort distribution a stratified random technique was employed, with a greater sampling effort in those strata that presented conditions of specific environments or microhabitats suitable for the presence of Theraphosidae and also to maximize the probability to inventory all species. It is important to mention that it has been observed that local distribution of Theraphosidae population can be fragmented in other areas of the country and that even though the sampling sites are adequated, these spiders may not be registered. For this reason, it is recommended to perform samplings in more extensive areas and thus increase the possibility of registering these fragment distributions.

Although possible errors associated with the species accumulation curve were considered, an initial exponential growth was not possible, neither an asymptotic number of species. This fact has been observed in other studies of spider diversity, in which the quality of spider inventory is evaluated and subsequently it is observed that it is not possible to register all species, causing accumulation curves to vary greatly from the asymptote (Brennan, Majer, & Reygaert, 1999; Coddington, Young, & Coyle, 1996; Edwards, 1993; Jimenez‐Valverde & Hortal, 2003; Samu & Lövei, 1995; Sorensen et al., 2002; Toti, Coyle, & Miller, 2000).

The present work confirms the presence of *G. rosea* in the Metropolitan Region, Chile, specifically in the eastern zone in the Andean foothills and associated with a type of sclerophyllous forest vegetation with *Acacia* scrub. The largest number of individuals was found on the slopes or plains at low altitude, less than 700 meters above sea level, with population numbers decreasing as altitude increases (see altitude in data accessibility). *G. rosea* has been cited for several cities in Chile, in the northern zone of the IV Region of Coquimbo (Coquimbo), central zone for the V Region of Valparaíso (Valparaíso) and Metropolitan Region (Santiago), and in the southern zone from the VI Region to the VIII Region of Bio Bio(Talcahuano) (Aguilera, 2015; Legendre & Calderón, 1984; Pocock, 1903). This work establishes the northern limit of the 28.5°S Atacama Region (Vallenar) and the southern limit of 37.8°S of the Biobio Region (Angol) for the studied species. This wide distribution of *G. rosea* facilitates evaluating the state of the different populations throughout the country, with studies of the genetic structure of each population, to analyze the distribution of inter‐ and intranatural population variability. Additionally, future studies could examine the processes involved, to later infer regarding relationships among different entities, as well as deterministic evolutionary mechanisms (natural selection, migration and mutation) and stochastic (genetic drift), as well as demographic processes (changes in population size) involved in the maintenance of variability in a certain group of organisms. This context could facilitate interpretation of the adaptive strategies of these spiders or be used to implement management units in biodiversity conservation plans (Remis, 2011), and also analyze phylogenetic aspects to better understand the presence of this genus in Chile.

The methodology proposed here could be used in future records of Theraphosidae and especially for environmental impact studies and environmental baselines.

## CONFLICT OF INTEREST

None declared.

## AUTHOR CONTRIBUTIONS

MAA and RM conceived the ideas and did the field trip and collect data; MAA and MEC designed methodology and analyzed the data. All authors wrote the manuscript and contributed critically to the drafts and gave final approval for publication.

## Data Availability

Data available from Figshare Knowledge Repository: https://doi.org/10.6084/m9.figshare.6493454.v1.
